# Age-standardized expected years of life lost: quantification of cancer severity

**DOI:** 10.1186/s12889-019-6843-9

**Published:** 2019-05-02

**Authors:** Yueh Wang, Chun-Ju Chiang, Wen-Chung Lee

**Affiliations:** 1 Taiwan Cancer Registry, Rm 506, No. 17, Xuzhou Rd., Taipei, 100 Taiwan; 2 Institute of Epidemiology and Preventive Medicine, College of Public Health, National Taiwan University, Taipei, Taiwan; 3 Innovation and Policy Center for Population Health and Sustainable Environment, College of Public Health, National Taiwan University, Taipei, Taiwan

**Keywords:** Expected years of life lost, Age-standardization, Cancer, Burden of disease

## Abstract

**Background:**

The critical implications of the expected years of life lost (EYLL) index of cancer for health policy assessments have been largely overlooked. We advocate to standardize life lost indices.

**Methods:**

Using the Taiwan Cancer Registry database as an example, we calculated the EYLL and the age-standardized EYLL to facilitate comparisons among cancer types (a total of 903,935 patients from the database). The International Cancer Survival Standard was used for calculating age-standardized EYLL.

**Results:**

Pancreatic cancer is the most severe cancer in Taiwan, with the greatest age-standardized EYLL for the men (15.6 years) and women (18.0 years) as well as for the men and women combined (16.6 years). Negative correlations were observed between unstandardized EYLL of cancer and mean age at diagnosis.

**Conclusions:**

The unstandardized EYLL represents an overall assessment of disease burden, whereas the age-standardized EYLL is a suitable measure of disease severity. We suggest that both measures be incorporated into routine annual reports of cancer statistics alongside the usual incidence and mortality rates and their age-standardized counterparts.

**Electronic supplementary material:**

The online version of this article (10.1186/s12889-019-6843-9) contains supplementary material, which is available to authorized users.

## Background

Cancer is a major public health problem worldwide; 17.5 million incident cases and 8.7 million mortality cases were reported for 2015 [1–7]. Cancer incidence and mortality rates and their age-standardized counterparts are used as references in cancer surveillance and cancer control in many countries [2, 8–13].

Expected years of life lost (EYLL) [14] is an alternative measure of the disease burden and economic effect. The EYLL of a patient with cancer can be interpreted as the patient’s average deprivation of life due to cancer or the average life years that could be saved if the patient had not developed cancer [1, 15]. Economic effect such as lifetime cost can also be derived via the EYLL and reimbursement database (for example, National Health Insurance Research Database in Taiwan) [15]. Therefore the EYLL is a useful index that reflects different aspects of health outcome. Recently, several studies have evaluated disease burdens from the EYLL perspective [15–22]. However, compared with the widely used and well-recognized indices of incidence and mortality rates, the critical nature of the EYLL index for health policy assessments has been largely overlooked.

In this paper, we advocate to standardize life lost indices. In the study reported herein, the age-standardized EYLL facilitated the comparisons of disease severity between cancer types and populations. Data regarding 20 major cancers from the Taiwan Cancer Registry were used in an example analysis.

## Methods

We chose 20 cancer types listed as follows (in alphabetical order): bladder, brain, bronchus and lung, cervix uteri, colon, corpus uteri, esophagus, female breast, kidney, leukemia, liver, nasopharynx, non-Hodgkin lymphoma, oral cavity, ovary, pancreas, prostate, rectum, stomach, and thyroid (corresponding International Classification of Diseases for Oncology, Third Edition (ICD-O-3) codes and morphology codes are presented in Additional file 1: Table S1). In total, 903,935 patients were identified from the National Cancer Registry Database of the Taiwan Cancer Registry. The patients were aged more than 15 years and had received their diagnoses between 2006 and 2015. The patients were followed up until their deaths or until December 31, 2016, whichever came first.

Hwang and Wang’s method was used to calculate the EYLL [14]. Specifically, the following four steps were performed.

1. For every patient under each type of cancer, we used the Monte Carlo method and the life table of the general population in Taiwan to randomly generate a survival time based on the patient‘s age, sex, and the year of diagnosis. For some rare cancers, we randomly generated more than one survival time for a patient, such that the total number of generated survival times was not less than 100,000. We then used the Kaplan–Meier method to obtain the survival curve for the generated reference population. The starting time of this survival curve was the time that the corresponding patient received the cancer diagnosis.
2. We calculated the survival curve of the patient population by using the same Kaplan–Meier method. The starting time of the survival curve was also the time at which the cancer was diagnosed.
3. For each time point *t*, the survival ratio between the patient population and reference population, *W*(*t*), was assumed to be between 0 and 1. We fit a restricted cubic spline model to logit[*W*(*t*)] and then extrapolated the function to *t* = 80. The function was subsequently back transformed into the original *W*(*t*) function. Multiplying the survival function of the reference population from step 1 by the extrapolated *W*(*t*), we obtained the extrapolated survival of the patient population.
4. The EYLL was calculated as the difference between the area under the survival curve of the reference population from step 1 and that under the survival curve of the patient population from step 2 (actual follow-up) and step 3 (extrapolation).

The bootstrap method was used to obtain the standard error of the EYLL with a resampling of 100 times. We used iSQoL2 statistical package R (http://sites.stat.sinica.edu.tw/isqol/) for the aforementioned computations.

The International Cancer Survival Standard (ICSS) [23] was used for age standardization of the EYLL. ICSS was formulated using the population-based cancer registry data from the European Cancer Registry Based Study on Survival and Care of Cancer Patients (EUROCARE) project (the project is now based on 22 countries in Europe [24]). The Surveillance, Epidemiology, and End Results Program of the United States has also suggested using the ICSS as the standard for cancer survival analysis.

## Results

As indicated in Table 1, the unstandardized EYLLs for the following three cancer types were greater than 15 years for the men and women combined: brain (22.5 years), esophagus (19.0 years), and pancreas (15.2 years). The data in Table 2 indicate that for the men, the unstandardized EYLLs for the following four cancer types were greater than 15 years: brain (20.0 years), esophagus (19.3 years), pancreas (15.2 years), and liver (15.2 years). Additionally, as indicated in Table 3, the unstandardized EYLLs for the following three cancer types were greater than 15 years for the women: brain (26.0 years), pancreas (15.8 years), and esophagus (15.0 years).

**Table 1:** Unstandardized EYLLs and age-standardized EYLLs of 20 cancers in men and women combined in Taiwan (standard error in parenthesis)

Cancer type	Number of patients	Mean age at diagnosis	Unstandardized		Age-standardized	
			EYLL (SE)	Rank	EYLL (SE)	Rank
Pancreas	17,994	67.6	15.2 (0.2)	3	16.6 (0.2)	1
Brain	6929	49.4	22.5 (1.5)	1	15.7 (0.3)	2
Esophagus	22,244	58.9	19.0 (0.4)	2	14.6 (0.1)	3
Bronchus and lung	109,075	68.0	13.0 (0.2)	7	14.0 (0.1)	4
Liver	112,904	64.5	14.7 (0.1)	4	14.0 (0.1)	5
Leukemia	18,120	57.4	11.9 (0.7)	8	11.0 (0.3)	6
Stomach	37,994	68.2	9.6 (0.3)	10	10.6 (0.2)	7
Ovary	12,308	52.0	13.7 (1.3)	6	10.5 (0.5)	8
Oral cavity	62,406	55.0	14.0 (0.3)	5	9.8 (0.2)	9
Nasopharynx	15,699	51.0	10.5 (0.9)	9	8.0 (0.3)	10
Non-Hodgkin	17,615	61.9	8.7 (0.7)	11	7.7 (0.3)	11
Kidney	10,812	61.2	6.9 (0.8)	12	7.1 (0.6)	12
Colon	79,349	66.6	6.3 (0.2)	14	6.5 (0.1)	13
Rectum	53,339	64.7	6.7 (0.3)	13	6.3 (0.2)	14
Bladder	20,920	69.6	5.3 (0.3)	18	5.6 (0.3)	15
Cervix uteri	16,665	57.4	5.9 (0.3)	16	5.5 (0.2)	16
Corpus uteri	17,336	54.3	5.4 (0.9)	17	5.1 (0.4)	17
Female breast	96,204	53.9	6.0 (0.5)	15	4.3 (0.2)	18
Prostate	43,320	73.2	2.8 (0.2)	19	3.4 (0.5)	19
Thyroid	26,081	48.4	2.1 (1.5)	20	3.0 (0.8)	20
All cancers irrespective of types	903,935	62.3	9.8 (0.1)	–	8.9 (0.1)	–

**Table 2:** Unstandardized EYLLs and age-standardized EYLLs of 16 cancers in men in Taiwan (standard error in parenthesis)

Cancer type	Number of patients	Mean age at diagnosis	Unstandardized		Age-standardized	
			EYLL (SE)	Rank	EYLL (SE)	Rank
Pancreas	10,224	66.7	15.2 (0.2)	3	15.6 (0.2)	1
Esophagus	20,745	58.4	19.3 (0.3)	2	14.6 (0.1)	2
Brain	3923	49.5	20.0 (1.8)	1	14.5 (0.3)	3
Bronchus and lung	67,687	69.0	12.2 (0.2)	6	13.8 (0.1)	4
Liver	78,676	62.6	15.2 (0.1)	4	13.4 (0.1)	5
Stomach	23,977	69.0	9.2 (0.3)	9	10.5 (0.3)	6
Leukemia	10,695	58.3	11.1 (0.8)	7	10.1 (0.4)	7
Oral cavity	56,946	54.5	14.6 (0.3)	5	10.0 (0.2)	8
Nasopharynx	11,882	51.1	10.2 (0.8)	8	8.0 (0.4)	9
Non-Hodgkin	9669	62.3	8.6 (0.9)	10	7.5 (0.4)	10
Rectum	32,286	64.8	6.6 (0.3)	11	6.1 (0.2)	11
Kidney	7136	60.8	5.7 (0.7)	13	6.1 (0.6)	12
Colon	43,822	66.8	6.1 (0.3)	12	6.0 (0.2)	13
Bladder	14,869	69.5	4.3 (0.4)	14	4.6 (0.3)	14
Thyroid	6188	49.9	4.1 (2.7)	15	4.1 (0.8)	15
Prostate	43,320	73.2	2.8 (0.2)	16	3.4 (0.5)	16
All cancers irrespective of types	502,842	63.7	10.6 (0.1)	–	9.7 (0.1)	–

**Table 3:** Unstandardized EYLLs and age-standardized EYLLs of 19 cancers in women in Taiwan (standard error in parenthesis)

Cancer type	Number of patients	Mean age at diagnosis	Unstandardized		Age-standardized	
			EYLL (SE)	Rank	EYLL (SE)	Rank
Pancreas	7770	68.7	15.8 (0.4)	2	18.0 (0.4)	1
Brain	3006	49.1	26.0 (2.1)	1	17.1 (0.5)	2
Liver	34,228	68.8	13.5 (0.2)	6	15.1 (0.3)	3
Esophagus	1499	66.0	15.0 (1.0)	3	15.0 (0.4)	4
Bronchus and lung	41,388	66.2	14.3 (0.3)	4	14.3 (0.2)	5
Leukemia	7425	56.1	13.5 (1.3)	7	12.0 (0.5)	6
Stomach	14,017	66.8	10.1 (0.4)	10	10.8 (0.3)	7
Ovary	12,308	52.0	13.7 (1.3)	5	10.5 (0.5)	8
Kidney	3676	61.8	10.6 (1.6)	9	9.2 (1.0)	9
Nasopharynx	3817	50.7	11.5 (2.5)	8	8.6 (0.8)	10
Bladder	6051	70.0	7.7 (0.6)	13	8.5 (0.6)	11
Non-Hodgkin	7946	61.5	8.6 (0.9)	12	7.8 (0.6)	12
Oral cavity	5460	60.4	9.2 (1.2)	11	7.7 (0.5)	13
Colon	35,527	66.4	6.2 (0.3)	15	7.0 (0.2)	14
Rectum	21,053	64.6	7.0 (0.4)	14	6.9 (0.4)	15
Cervix uteri	16,665	57.4	5.9 (0.3)	17	5.5 (0.2)	16
Corpus uteri	17,336	54.3	5.4 (0.9)	18	5.1 (0.4)	17
Female breast	96,204	53.9	6.0 (0.5)	16	4.3 (0.2)	18
Thyroid	19,893	47.9	1.5 (2.1)	19	3.6 (1.1)	19
All cancers irrespective of types	401,093	60.4	8.6 (0.1)	–	8.0 (0.1)	–

As shown in Table 1, the age-standardized EYLLs for the pancreas (16.6 years) and brain (15.7 years) were greater than 15 years for the men and women combined. Additionally, the EYLL associated with cancer of the pancreas (15.6 years) was greater than 15 years for the men (Table 2). As denoted in Table 3, four cancer types with age-standardized EYLLs greater than 15 years were observed for the women: pancreas (18.0 years), brain (17.1 years), liver (15.1 years), and esophagus (15.0 years). The unstandardized and the age-standardized EYLLs for all cancers were also presented in Table 1 (male and female combined), Table 2 (male), and Table 3 (female), respectively. Note that these include other minor cancer types that were not shown in this study.

Notable differences were identified between the unstandardized and age-standardized EYLLs for several cancer types. As indicated in Tables 1 and 2, the unstandardized EYLLs for cancer of the oral cavity in the men (14.6 years) as well as in the men and women combined (14.0 years) were greater than those for cancer of the bronchus and lung (12.2 years and 13.0 years, respectively). However, the converse was true after age standardization. The age-standardized EYLLs for cancer of the oral cavity in the men (10.0 years) as well as in the men and women combined (9.8 years) were smaller than those associated with cancer of the bronchus and lung (13.8 years and 14.0 years, respectively). In addition, the unstandardized EYLLs for cancer of the stomach in the men (9.2 years), women (10.1 years), and men and women combined (9.6 years) ranked in the middle among the 20 cancer types (the ninth, tenth, and tenth places, respectively), whereas the rankings of the corresponding age-standardized EYLLs (10.5, 10.8, and 10.6 years, respectively) were sixth, seventh, and seventh, respectively (Tables 1, 2 and 3). The rankings of the unstandardized EYLLs for cancer of the bronchus and lung in the men (12.2 years) and in the men and women combined (13.0 years) were sixth and seventh, respectively, whereas the rankings of the corresponding age-standardized EYLLs (13.8 and 14.0 years, respectively) were both fourth. The ranking of the unstandardized EYLL for cancer of the liver in the women (13.5 years) was sixth, but the ranking of its age-standardized EYLL (15.1 years) was third.

**Fig. 1:**
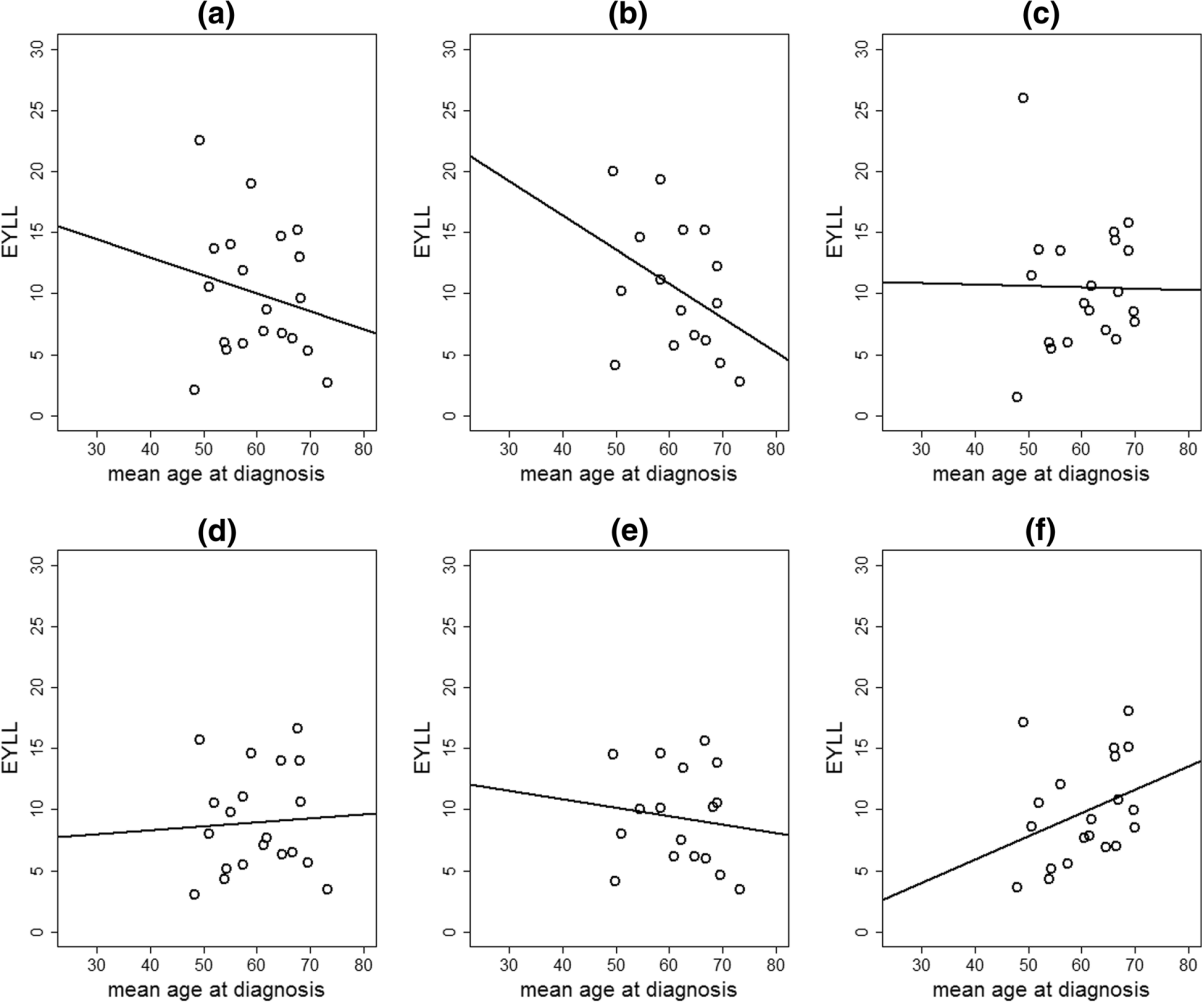
Scatterplots of mean ages at diagnoses and the unstandardized expected years of life lost (EYLL) associated with 20 cancers in men and women combined (**a**), 16 cancers in men (**b**), and 19 cancers in women (**c**), as well as the age-standardized EYLL associated with 20 cancers in men and women combined (**d**), 16 cancers in men (**e**), and 19 cancers in women (**f**)

Negative correlations of moderate magnitudes were observed between the unstandardized EYLLs and the mean corresponding patient ages at the time of diagnosis among 20 cancers in the men and women combined (Fig. 1a, correlation coefficient = − 0.20), among 16 cancers in men (Fig. 1b, correlation coefficient = − 0.39), and among 19 cancers in women (Fig. 1c, correlation coefficient = − 0.02). These results indicated that a larger unstandardized EYLL for a cancer type may have been due to a younger mean age at diagnosis rather than greater severity. After age standardization, the negative correlations disappeared (Fig. 1d, correlation coefficient = 0.06; Fig. 1f, correlation coefficient = 0.34) or diminished in magnitude (Fig. 1e, correlation coefficient = − 0.13). After adjusting the confounding effect of age, the age-standardized EYLL properly reflected the severity of the corresponding cancer type.

## Discussion

Unstandardized EYLLs [16, 21, 22] were confounded by age at diagnosis and cancer severity. A patient with a cancer associated with relatively favorable prognosis may have a higher EYLL if the patient is of a relatively young age at the time of diagnosis. For example, cancer of the oral cavity (a cancer with a relatively favorable prognosis) in the men and women combined has the younger mean age at diagnosis of 55.0 years and greater unstandardized EYLL of 14.0 years than the corresponding figures for cancer of the bronchus and lung (a cancer associated with bleak prognosis) in the men and women combined (mean age at diagnosis: 68.0; unstandardized EYLL: 13.0 years). Therefore, the unstandardized EYLL may be used to represent overall disease burden, but it is not a suitable measure of disease severity.

The literature lacks studies that have applied age standardization in calculations of EYLL. This study fills that gap. Age-standardized EYLL may be used to facilitate the comparisons of the severities of cancer types. For example, pancreatic cancer corresponded with the greatest age-standardized EYLL in the men and women combined in Taiwan, indicating that patients with pancreatic cancer are likely to undergo a greater deprivation of life than did patients who receive diagnoses of other cancer types at the same age as the patients with pancreatic cancer receive their diagnoses. Age standardization also enables comparison of the severity of a cancer type among countries. The World Health Organization (WHO) World Standard Population [25] is widely used in age standardization of indices based on general populations, such as indices for incidence and mortality rates [26]. By contrast, ICSS population is used in age standardization for indices based on patient populations, such as indices for survival proportion [27]. In this study, the EYLL was calculated based on patient population; therefore, ICSS was suitable for population age standardization. ICSS populations differ among cancer types: ICSS1 corresponds with cancers that increase in incidence as population age increases; ICSS2 corresponds with cancers that are broadly constant in incidence in relation to population age; and ICSS3 corresponds with cancers that mainly affect young adults [23]. To compare the severity of a specific cancer among countries or regions, the corresponding ICSS standard population for age standardization should be used. However, according to the concept behind the “identical weighting system” [28, 29], use of equal standard populations in comparisons of cancer types in a country or region is crucial for eliminating the confounding effect of age. Therefore, in this study, we used ICSS1 for severity comparisons among all cancer types.

From the individual perspective, it is appropriate to apply the age-specific EYLL to make inference about the life lost. We also calculated the age-specific EYLLs (5 strata: 15–44, 45–54, 55–64, 65–74, and > 75 years) associated with 20 cancers for the men and women combined, 16 cancers for the men, and 19 cancers for the women (Additional file 1: Tables S2–S4). Based on these results, a cancer patient in Taiwan can then be informed of his or her EYLL in accordance with age at the time of diagnosis and the type of cancer diagnosed. For example, a 60-year-old man diagnosed with oral cancer will have an EYLL of 11.9 years (Additional file 1: Table S3), and a 50-year-old woman diagnosed with cervical cancer will have an EYLL of 7.7 years (Additional file 1: Table S4).

Another well-known measure to evaluate cancer severities is the relative survival [30], which is the survival under a hypothetical situation that the disease of interest is the only cause of death. A relative survival is predicated on a given cut point of time, for example, the 3-year or 5-year relative survival; information beyond that time point is totally discarded. By comparison, the EYLL is a complete follow-up of the expected life-time loss, not just limited to a cut-off time point. Moreover, the concept behind the EYLL may be helpful in better communicating cancer severity to the general public.

In addition to age standardization achieved using standard populations (such as the WHO standard population or the ICSS population), the lifetime cumulative sum of age-specific rates (or the weighted sum where life-table survival function or potential life lost is used as the weight) by itself may be used as an age-standardized index. For example, the cumulative rate of potential life lost and the lifetime years of potential life lost, previously proposed by Lee [31, 32], are age-standardized indices and can be interpreted as the expected years of potential life lost due to a specific cause of death during the lifetime of an individual. Such lifetime age standardization represents an alternative perspective from which to address the present EYLL problem and should be further studied. In this study, EYLL was determined using an incidence-based approach whereby patients with cancer were followed up for a period of time to monitor their survival statuses and the results regarding their lifespans were then extrapolated. By comparison, the years of potential life lost (YPLL) due to cancer is calculated using only the information of those who have died from cancer [21]. An additional advantage of the incidence-based approach is that it does not include cause-of-death information; therefore, it is immune to coding errors associated with calculations based on underlying causes of death. Finally, in this study, we used a restricted cubic spline model [14] for lifespan extrapolation. The “cure models” [33–35], which assume that excess hazard due to cancer decreases toward zero over time, are alternative (and potentially superior) methods for extrapolation.

## Conclusion

The EYLL and age-standardized EYLL reveal disease burden and disease severity, respectively, which are critical measures from population-wide as well as individual perspectives. We suggest that both measures be incorporated into routine annual reports of cancer statistics alongside the usual measures of incidence and mortality rates and their age-standardized counterparts.

## Additional file

Additional file 1: **Table S1.** ICD-O-3 code and morphological code used in this study. **Table S2.** Age-specific EYLLs for 20 major cancers in men and women combined in Taiwan. **Table S3.** Age-specific EYLLs for 16 major cancers in men in Taiwan. **Table S4.** Description of data: Age-specific EYLLs for 19 major cancers in women in Taiwan. (DOCX 34 kb)

## Abbreviation

EYLL: Expected years of life lost
