# Supplementary material for: Age-standardized expected years of life lost: quantification of cancer severity
Source: BMC Public Health. 2019 May 2;19:486. doi: 10.1186/s12889-019-6843-9 (PMC6498471; doi:10.1186/s12889-019-6843-9)
Supplement: Supplementary file 1 — Table S1. ICD-O-3 code and morphological code used in this study. Table S2. Age-specific EYLLs for 20 major cancers in men and women combined in Taiwan. Table S3. Age-specific EYLLs for 16 major cancers in men in Taiwan. Table S4. Description of data: Age-specific EYLLs for 19 major cancers in women in Taiwan. (DOCX 34 kb) [file 12889_2019_6843_MOESM1_ESM.docx]

**Additional file 1: Table S1-S4**

**Table S1** ICD-O-3 code and morphological code used in this study

| Cancer type | ICD-O-3 code | Morphological code |
| --- | --- | --- |
| Bladder | C67 | - |
| Brain | C71 | - |
| Bronchus and lung | C33–C34 | - |
| Cervix uteri | C53 | - |
| Colon | C18 | - |
| Corpus uteri | C54 | - |
| Esophagus | C15 | - |
| Female breast | C50 | - |
| Kidney | C64 | - |
| Leukemia | - | 98663, 98713, 98963, 98973, 98953, 99203, 98613, 98403, 98673, 98703, 98723, 98733, 98743, 98913, 99103, 99313, 99303, 97273, 98353, 98363, 98373, 98633, 98753, 98763, 99453, 99463, 99633, 99643, 98233, 98013, 97401, 97403, 97413, 97423, 99503, 99603, 99613, 99623, 99753, 99803, 99823, 99833, 99843, 99853, 99863, 99873, 99893, 98053, 98203, 98323, 98603, 98003 |
| Liver | C22 | - |
| Nasopharynx | C11 | - |
| Non-Hodgkin lymphoma | - | 96893, 96993, 96713, 97613, 96753, 96903, 96913, 96953, 96983, 96733, 96873, 98263, 96803, 96843, 96793, 95963, 96703, 96783, 97283, 98333, 99403 |
| Oral and Pharynx | C00–C06, C09, C10, C12–C14 | - |
| Ovary (including fallopian tube and broad ligament) | C56, C570–C574 |  |
| Pancreas | C25 | - |
| Prostate | C61 | - |
| Rectum and Anus | C19–C21 | - |
| Stomach | C16 | - |
| Thyroid | C73 | - |

**Table S2** Age-specific EYLLs for 20 major cancers in men and women combined in Taiwan

| Cancer type | Age | | | | |
| --- | --- | --- | --- | --- | --- |
|  | 15–44 | 45–54 | 55–64 | 65–74 | >75 |
| Bladder | 9.2 | 6.4 | 7.4 | 5.4 | 3.2 |
| Brain | 35.8 | 23.8 | 18.5 | 13.3 | 7.5 |
| Female breast | 9.2 | 6.6 | 5.0 | 4.1 | 1.8 |
| Bronchus and lung | 29.6 | 23.5 | 16.9 | 11.5 | 6.6 |
| Cervix uteri | 8.8 | 7.7 | 6.1 | 5.2 | 3.7 |
| Colon | 16.9 | 9.8 | 7.4 | 5.0 | 3.4 |
| Corpus uteri | 9.8 | 5.0 | 6.6 | 4.7 | 3.3 |
| Esophagus | 31.7 | 24.8 | 18.3 | 11.5 | 6.4 |
| Kidney | 17.2 | 12.5 | 8.0 | 5.2 | 3.7 |
| Leukemia | 20.9 | 14.3 | 12.7 | 10.7 | 6.1 |
| Liver | 31.3 | 23.0 | 16.8 | 11.3 | 6.5 |
| Nasopharynx | 10.6 | 10.5 | 9.5 | 7.7 | 5.6 |
| Non-Hodgkin | 12.7 | 9.5 | 8.8 | 7.6 | 4.9 |
| Oral cavity | 21.1 | 16.5 | 11.7 | 7.8 | 4.8 |
| Ovary | 17.0 | 13.2 | 12.5 | 10.7 | 6.0 |
| Pancreas | 36.4 | 26.6 | 20.1 | 13.9 | 7.4 |
| Prostate | 12.0 | 5.0 | 3.5 | 2.4 | 1.6 |
| Rectum | 15.0 | 10.6 | 7.1 | 4.7 | 3.5 |
| Stomach | 24.0 | 18.7 | 12.3 | 8.1 | 5.1 |
| Thyroid | 1.4 | 5.0 | 1.8 | 3.7 | 3.0 |

**Table S3** Age-specific EYLLs for 16 major cancers in men in Taiwan

| Cancer type | Age | | | | |
| --- | --- | --- | --- | --- | --- |
|  | 15–44 | 45–54 | 55–64 | 65–74 | >75 |
| Bladder | 6.6 | 5.3 | 6.1 | 4.5 | 2.7 |
| Brain | 32.4 | 22.6 | 17.4 | 12.1 | 7.1 |
| Bronchus and lung | 29.5 | 23.1 | 16.4 | 11.3 | 6.5 |
| Colon | 15.4 | 7.9 | 7.2 | 5.0 | 3.2 |
| Esophagus | 32.1 | 24.5 | 18.4 | 11.6 | 6.3 |
| Kidney | 15.8 | 10.5 | 6.1 | 5.0 | 3.0 |
| Leukemia | 18.0 | 13.4 | 11.8 | 9.9 | 5.8 |
| Liver | 31.1 | 22.7 | 16.2 | 10.5 | 6.0 |
| Nasopharynx | 11.6 | 10.0 | 9.8 | 7.2 | 5.6 |
| Non-Hodgkin | 10.6 | 9.3 | 9.9 | 7.4 | 4.4 |
| Oral cavity | 21.6 | 16.6 | 11.9 | 7.9 | 4.9 |
| Pancreas | 34.9 | 25.4 | 18.9 | 12.9 | 7.0 |
| Prostate | 12.0 | 5.0 | 3.5 | 2.4 | 1.6 |
| Rectum | 13.3 | 10.1 | 7.1 | 4.6 | 3.5 |
| Stomach | 25.3 | 18.0 | 12.2 | 8.1 | 4.9 |
| Thyroid | 0.8 | 2.5 | 5.4 | 5.7 | 3.1 |

**Table S4** Age-specific EYLLs for 19 major cancers in women in Taiwan

| Cancer type | Age | | | | |
| --- | --- | --- | --- | --- | --- |
|  | 15–44 | 45–54 | 55–64 | 65–74 | >75 |
| Bladder | 17.8 | 10.6 | 10.7 | 7.6 | 4.6 |
| Brain | 39.2 | 25.2 | 20.3 | 14.9 | 8.0 |
| Female breast | 9.2 | 6.6 | 5.0 | 4.1 | 1.8 |
| Bronchus and lung | 30.3 | 23.5 | 17.4 | 11.7 | 6.8 |
| Cervix uteri | 8.8 | 7.7 | 6.1 | 5.2 | 3.7 |
| Colon | 18.6 | 12.2 | 7.5 | 5.0 | 3.6 |
| Corpus uteri | 9.6 | 5.0 | 6.5 | 4.7 | 3.4 |
| Esophagus | 29.1 | 27.1 | 18.4 | 12.2 | 6.6 |
| Kidney | 20.4 | 16.4 | 11.9 | 5.5 | 5.1 |
| Leukemia | 23.0 | 15.2 | 14.1 | 12.0 | 6.5 |
| Liver | 30.6 | 24.6 | 18.6 | 12.6 | 7.2 |
| Nasopharynx | 9.6 | 12.6 | 8.7 | 9.5 | 5.7 |
| Non-Hodgkin | 15.3 | 9.4 | 7.3 | 8.0 | 5.6 |
| Oral cavity | 11.4 | 11.3 | 10.0 | 6.6 | 4.6 |
| Ovary | 16.9 | 13.2 | 12.5 | 10.7 | 6.0 |
| Pancreas | 38.4 | 28.9 | 22.3 | 15.3 | 7.9 |
| Rectum | 20.5 | 11.3 | 7.2 | 4.9 | 3.6 |
| Stomach | 25.1 | 19.2 | 12.4 | 8.0 | 5.4 |
| Thyroid | 10.3 | 4.8 | 1.7 | 3.4 | 3.2 |
